# Type 2 diabetes prevalence, awareness, and risk factors in rural Mali: a cross-sectional study

**DOI:** 10.1038/s41598-023-29743-1

**Published:** 2023-03-06

**Authors:** Abdoulaye Diawara, Djibril Mamadou Coulibaly, Talib Yusuf Abbas Hussain, Cheickna Cisse, Jian Li, Mamadou Wele, Mahamadou Diakite, Kassim Traore, Seydou O. Doumbia, Jeffrey G. Shaffer

**Affiliations:** 1 University of Sciences, Techniques and Technologies of Bamako, Bamako, Mali; 2 Burhani College, Mazgaon, Mumbai India; 3 Campbell University, Buies Creek, NC USA; 4 Tulane University School of Public Health and Tropical Medicine, New Orleans, LA USA

**Keywords:** Epidemiology, Diabetes, Pre-diabetes, Risk factors

## Abstract

Diabetes is currently a crisis in sub-Saharan West Africa (SSWA) with dramatic implications for public health and national budgets prioritizing infectious diseases. There is limited recent literature about the prevalence, awareness, and risk factors for type 2 diabetes (T2D) in rural parts of SSWA. This study characterized T2D prevalence and risk factors for the rural Malian community of Nièna, which is situated in Mali’s second-largest province of Sikasso. Between December 2020 and July 2021, a cross-sectional study of 412 participants was conducted in the Nièna community using clinical questionnaires and rapid diagnostic tests. Among 412 participants, there were 143 (34.7%) and 269 (65.3%) males and females, respectively. The overall prevalence of T2D in Nièna was 7.5% (31/412), and prevalence rates were 8.6% (23/269) and 5.6% (8/143) for females and males, respectively. Age, family history of diabetes, hypertension, waist circumference, and fetal macrosomia were significantly associated with T2D (p = 0.007, p < 0.001, p = 0.003, p = 0.013, and p < 0.001, respectively). Notably, 61.3% (19/31) of T2D subjects were unaware of their diabetic status before the study. Field surveys have considerable utility in driving T2D awareness in rural African settings.

## Introduction

According to the International Diabetes Federation (IDF), approximately 537 million people worldwide have diabetes, and there will be 783 million diabetics worldwide by 2045^1^. Over the past three decades, reported type 2 diabetes (T2D, commonly known as adult-onset diabetes) cases have risen dramatically and now account for approximately 90% of all diabetes cases^2^. Undiagnosed or inadequately controlled T2D poses risks of incapacitating complications such as damage to the heart, eyes, kidneys, and nerves. Diabetes is a major cause of blindness, kidney failure, heart attacks, stroke, and lower limb amputation^3,4^. Diabetic complications may also lead to an increased risk of vision loss and early death^5^.

While premature mortality from most major noncommunicable diseases (NCDs) has diminished over the past few decades, early death rates from diabetes increased by 5% between 2000 and 2016^5^. Increased diabetes prevalence poses significant impediments for countries in attaining goals such as those set forth by the United Nations Sustainable Development Goals^5^. Global studies have also shown an increased risk of severe illness and death among patients with coronavirus disease 2019 (COVID-19)^6,7^. A recent study showed that 18.3% of COVID-19 deaths in Africa were among people with diabetes, and nearly 20% of COVID-19 deaths were linked with diabetes^6–8^.

Diabetes is now widely recognized as a crisis in Africa^9^. Already maintaining the world’s most considerable burden of HIV/AIDS and malaria, Africa is now faced with alarming increases in diabetes^8,10^. Poverty, social inequality, and limited access to quality health care have exasperated the diabetes epidemic in Africa. Through the middle of the 1980s, diabetes was considered rare in sub-Saharan Africa, with reported prevalence rates in several countries reportedly less than 1%^11^. Africa has since experienced a sixfold increase in diabetes cases, rising from 4 million cases in 1980 to 25 million cases in 2014^2,6^. Diabetes prevalence in sub-Saharan West Africa (SSWA) has particularly increased over the past decade, affecting people in all sectors of society with substantial economic effects^3,4,8^. Health systems in SSWA frequently have inadequate capacity for mitigating the current burden of diabetes and its complications^9,12,13^. One study in Kenya revealed that 60% of people diagnosed with diabetes were not receiving treatment^14^.

Few recent studies have quantified the prevalence of diabetes in rural African communities, but those conducted have generally shown increased occurrence and low awareness levels. For instance, a cross-sectional study among adults in 24 communities from Zambia and South Africa reported age-standardized prevalence of diabetes rates of 3.5% and 7.2%, respectively^10^. This study also revealed that 34.5% of subjects in Zambia were unaware of their positive diabetic status. In Zambia, diabetic awareness was found to be higher among individuals with increased education and higher household socioeconomic position^10^. In Nigeria, a systematic review provided a comprehensive report on the epidemiology of T2D since its last nationwide NCD survey in 1997. Age-adjusted prevalence rates of T2D in Nigeria among persons aged 20–79 years increased from 2.0% in 1990 (874,000 cases) to 5.7% in 2015 (4.7 million cases)^15^. Additionally, many persons living with T2D were undiagnosed, and few reportedly received treatment^14,15^. T2D increases in Africa may be associated with factors such as urbanization, adaptative lifestyle behaviors (obesity, alterations in eating habits, and reduced physical activity), genetics^15^, hypertension, age demographics, and pregnancy rates^16,17^. A study in Kenya linked health-seeking behaviors in T2D subjects with treatment facility type, self-rated health status, alcohol use, hospital admission, and social support^18^. It was estimated that 59.7% of diabetes cases are undiagnosed in Africa (the highest percentage of undiagnosed cases in the world) and that higher proportions of undiagnosed cases are found in low-income countries than in middle-income countries^19^.

In 2021, T2D prevalence in Mali was reported as 1.8%^1^. Reported T2D prevalence rates for Mali between 1976 and 2017 have ranged from 0.4 to 5.0%. In 1976, T2D prevalence in Bamako was estimated at 1.4%, which was later estimated at 3.0% in 2017^16,20^. In a 1987 study at a referral center in Bamako, 0.9% of subjects with T2D resided in rural areas^12,14,21^. In 2017, a similar study approximately 90 miles from Bamako performed at a health center Sélingué health district reported T2D prevalence as 5.5%^16^. A recent study published in 2021 reported 1.7% T2D prevalence rates among 809 subjects surveyed in Bamako and six neighboring communes^22^.

The potential impact of T2D is substantial in Mali, with dramatic implications for public health and national budgets that largely prioritize infectious diseases^12,23^. The lack of literature and field studies in rural parts of the country has led to significant challenges in fighting the rise in T2D. To date, nationwide health surveys on T2D have not been routinely performed in Mali. While alarming increases in diabetes have been observed in Africa and urban locations in Mali, T2D has not been adequately quantified and characterized for rural Malian areas. It is therefore vital to determine and monitor the burden of diabetes in rural parts of Mali to facilitate appropriate health resource allocation, advocacy, and planning. Insight into the epidemiological aspects of T2D in rural communities is also needed to promote and provide better preventive and targeted therapeutic approaches. For these reasons, this study aimed to determine the prevalence, risk factors, and awareness of T2D in the rural Malian community of Nièna.

## Materials and methods

### Ethics statement

The study was approved by the Ethics Committee at the University of Sciences, Techniques and Technologies of Bamako (USTTB, reference number: 2021/164/CE/USTTB). All study participants were aged 20 years or older and provided oral informed consent prior to participation.

### Study site

**Figure 1:**
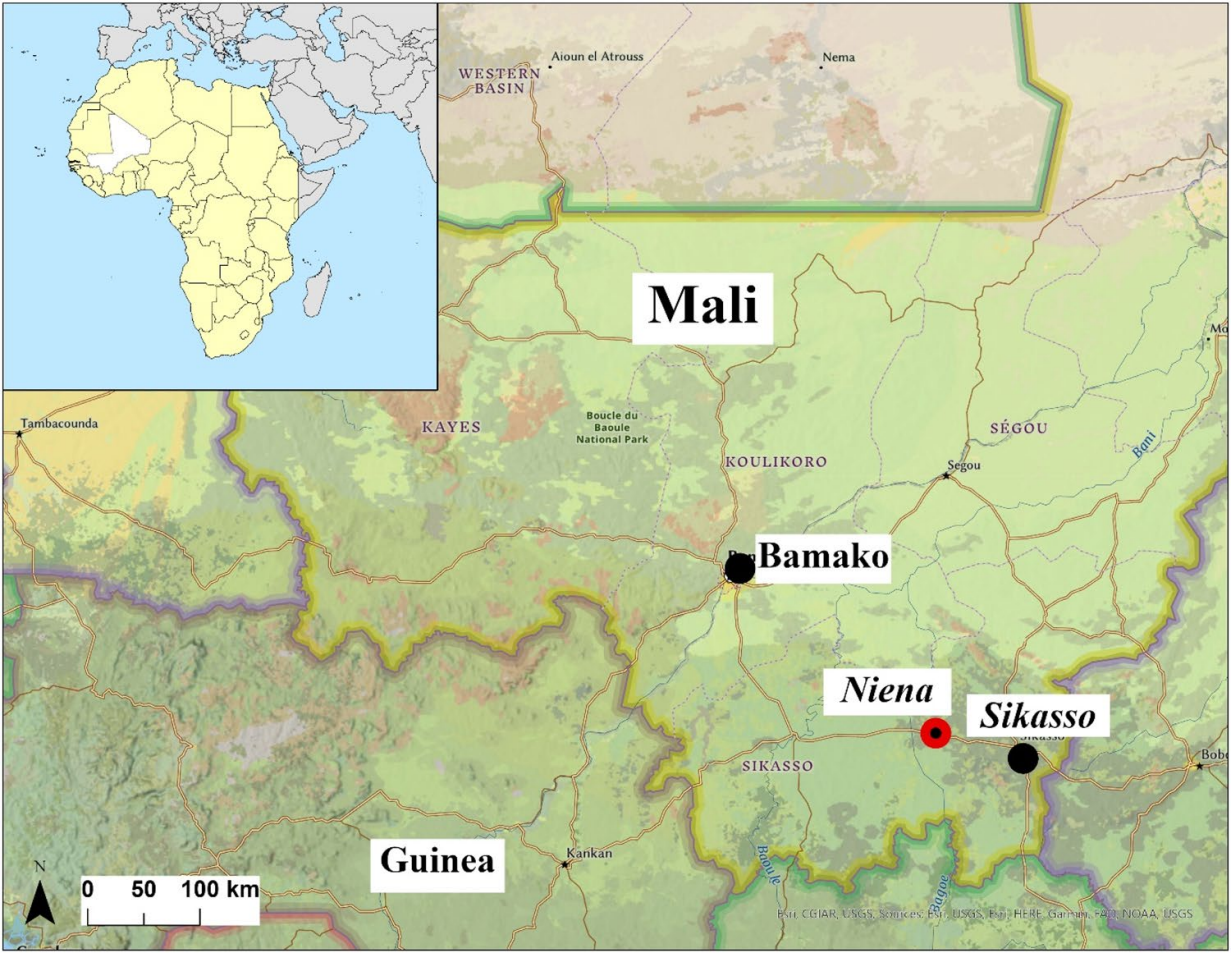
Geographical location of the study site in Niéna, Mali. Nièna is situated west of the Sikasso Region and southeast of the capital city of Bamako. The geographic coordinates for the locations shown are Bamako (12.6392° N, 8.0029° W), Sikasso (11.3224° N, 5.6984° W), and Nièna (11.4277° N, 6.3492° W). The map was generated using the ArcGIS application basemaps (Professonal version, ESRI, Redlands, CA). Source and service layer credits for satellite imagery: Esri, DigitalGlobe, GeoEye, i-cubed, USDA FSA, USGS, AEX, Getmapping, Aerogrid, IGN, IGP, swisstopo, and the GIS User Community.

Nièna is a rural commune in Mali, located in the Sikasso Circle along a national road (Highway RN7) connecting Bamako, Sikasso, and its neighboring country of Côte d’Ivoire^24^. The commune has an area of 1040 square kilometers, includes 45 villages, and a population of 51,086 (population density = 51,086/1040 = 49.1 people per square kilometer). This part of Mali is known for its subsistence agriculture in corn and rice^25^. Nièna is illustrated in terms of its proximity to Bamako in Fig. 1.

### Study design

This cross-sectional study was conducted between December 2020 and July 2021. Sample sizes were determined according to the estimated proportion of the population with T2D or pre-diabetes. The study sample size was determined based on a single proportion, specifically

$$n= {\left({z}_{1-\alpha/_2}\right)}^{2}\cdot p\left(1-p\right)/{d}^{2},$$

where *n* is the minimum sample size, α is the type I error rate, ${z}_{1-\alpha/_2}$ is the $1-\alpha /_2\mathrm{th}$ percentile for the standard normal distribution curve, and *d* is the margin of error. Assuming that the population living with T2D or pre-diabetes was 6%, for a type I error rate of 5%, a margin of error of 5%, and 10% non-response rate, the minimum sample size was estimated as 406 study subjects.

### Study population

Inclusion criteria were residency of the Nièna community age 20 years or older. Participants were recruited randomly through public health campaigns in Nièna and its surrounding communities of Dougoukolobougou and Banzana. Selected participants were consulted about the purpose of the study and informed of its potential risks. To foster community engagement and maximize study participation, outreach study personnel held meetings with leaders from villages, neighborhoods, religious groups, local health facilities, and administrative personnel before enrollment activities. All activities were undertaken following local community policies, regulations, and procedures.

### Data collection

Data were collected through individual interviews and clinical questionnaires, which captured information on sociodemographic characteristics (age, sex, occupation), ethnicity, and family health history of diabetes. Blood pressure and anthropometric characteristics, including height, body weight, and waist circumference, were measured and recorded. Blood tests were performed using finger pricks with Accu-Chek (Roche Diabetes Care, Inc., Indianapolis, IN) glucometers. Testing was based on fasting glycemia for participants fasting 8 to 12 h before testing.

### Definitions

Positive T2D status was defined according to IDF criteria as fasting plasma glucose of at least 126 mg/dL^1^. Patients were also considered as positive for T2D if they reported being previously diagnosed with T2D on the study questionnaire. Pre-diabetic status was classified as fasting plasma glucose between 111 and 125 mg/dL. Fasting plasma glucose levels under 111 mg/dL were considered non-diabetic. Participants with blood sugar levels of 111 mg/dL or higher were invited to complete a second test. Participants were categorized into four groups according to their body mass index (BMI) measurements. BMI break points were set at greater than or equal to 30.0 kg/m^2^ (obese), between 25.0 and 29.9 kg/m^2^ (overweight), between 18.5 and 24.9 kg/m^2^ (normal), and less than 18.5 kg/m^2^ (underweight). Abdominal obesity was defined as waist circumference of at least 80 cm and 90 cm for females and males, respectively. Hypertension was defined as systolic blood pressure 140 mmHg or higher, diastolic blood pressure of 90 mmHg or higher, or the current use of antihypertensive drugs. For family history of diabetes, immediate family members (father, mother, sister, or brother) were considered immediate family members.

### Statistical analysis

Data were expressed as frequencies and percentages. Univariate analyses were performed on the following predictors: sex, age, occupation, hypertension, family history of diabetes, BMI, waist circumference, and ethnicity. Pearson’s chi-square or Fisher’s Exact tests were used for comparing proportions between categorical variables. Pairwise comparisons between categorical predictors were performed using logistic regression models with multiple comparisons. Statistically significant risk factors in univariate analyses were included in multivariate logistic regression analyses (Supplemental Fig. S1). Data were analyzed using SPSS for Windows (version 26, IBM Corp., Armonk, NY) and the SAS System (version 9.4, SAS Institute, Inc., Cary, NC). The type I error threshold was set at 5%.

## Results

### Study population

A total of 412 subjects were enrolled, with 88.1% (363/412) classified as non-diabetic. The overall prevalence rates for pre-diabetes and diabetes were 4.4% (18/412) and 7.5% (31/412), respectively (Table 1).

**Table 1:** Baseline characteristics for n = 412 study participants, Nièna municipality, Mali, 2021.

Characteristic	Diabetic status			p value	
Age group, years	Non-diabetes (n = 363)	Pre-diabetes (n = 18)	Diabetes (n = 31)	General differences^5^	Non-diabetes vs. diabetes^6^
20–39	110 (30)	3 (17)	2 (6)	0.012	0.007
40–59	138 (38)	11 (61)	17 (55)		
≥ 60	115 (32)	4 (22)	12 (39)		
Sex				0.521	0.330
Female	235 (65)	11 (61)	23 (74)		
Male	128 (35)	7 (39)	8 (26)		
Ethnicity				0.481	0.306
Peulh	156 (43)	6 (33)	15 (48)		
Bambara	151 (42)	8 (44)	9 (29)		
Other	56 (15)	4 (22)	7 (23)		
Family history of T2D^1^				< 0.001	< 0.001
No	324 (89)	18 (100)	11 (35)		
Yes	39 (11)	0 (0)	20 (65)		
BMI (kg/m^2^)^2^				0.004	0.166
< 18.5	21 (6)	1 (6)	0 (0)		
18.5–24.9	188 (53)	2 (11)	12 (40)		
25.0–29.9	85 (24)	8 (44)	9 (30)		
≥ 30.0	64 (18)	7 (39)	9 (30)		
Hypertension^3^				0.002	0.003
No	289 (80)	11 (61)	17 (55)		
Yes	74 (20)	7 (39)	14 (45)		
Waist circumference^4^				0.036	0.013
Normal	153 (42)	6 (33)	6 (19)		
High	210 (58)	12 (67)	25 (81)		

Data are expressed as frequency (column percentage).

*BMI* body mass index, *T2D* type 2 diabetes.

^1^Based on immediate family members (father, mother, sister, or brother).

^2^BMI values were missing for five subjects in the non-diabetes group and one subject in the diabetes group.

^3^Hypertension defined as prior diagnosis of hypertension or systolic blood pressure greater than or equal to 140 mmHg or diastolic blood pressure of at least 90 mmHg or use of antihypertensive drugs.

^4^Normal defined as less than 80 cm and 90 cm for females and males, respectively. High was defined as at least 80 cm and 90 cm for females and males, respectively.

^5^Based on Fisher’s Exact Test for general differences among the diabetic groups.

^6^Based on Fisher’s Exact Test comparing the non-diabetes and diabetes groups.

### Sex and diabetic status

**Figure 2:**
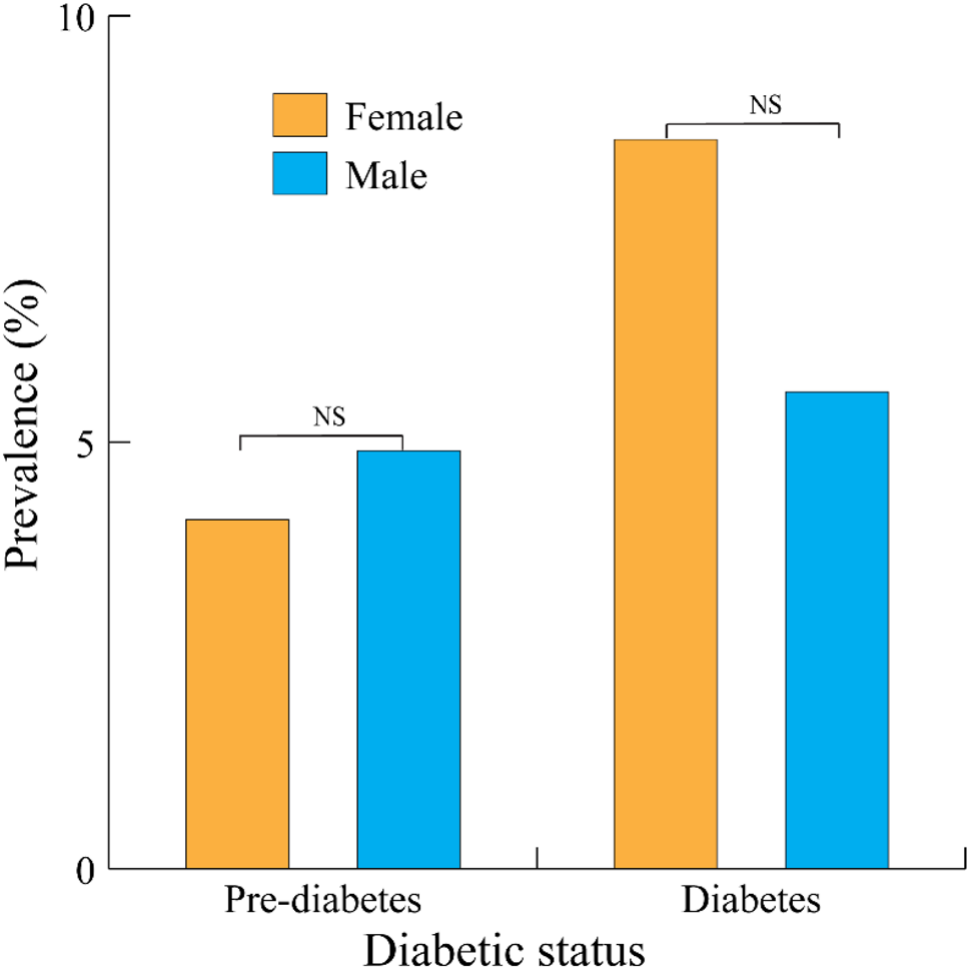
Type 2 diabetes prevalence by sex among n = 412 participants in Nièna, Mali, 2021. The first and second columns show the prevalence rates for the sampled female and male populations, respectively. Prevalence rates did not statistically differ by sex for either the pre-diabetic or diabetic groups.

Participants were 65.3% (269/412) female and 34.7% male (143/412). Approximately 57.0% (235/412) of participating subjects were non-diabetic females. The distribution of the sampled population by diabetic status and sex group is shown in Fig. 2.

Diabetes was more commonly observed in females than in males (8.6% [23/269] and 5.6% [8/143], respectively). Pre-diabetes was slightly more common among males than females (4.9% [7/143] and 4.1% [11/269], respectively). However, neither of these differences was statistically significant (p = 0.754 and p = 0.291, respectively).

### Age by diabetic status

**Figure 3:**
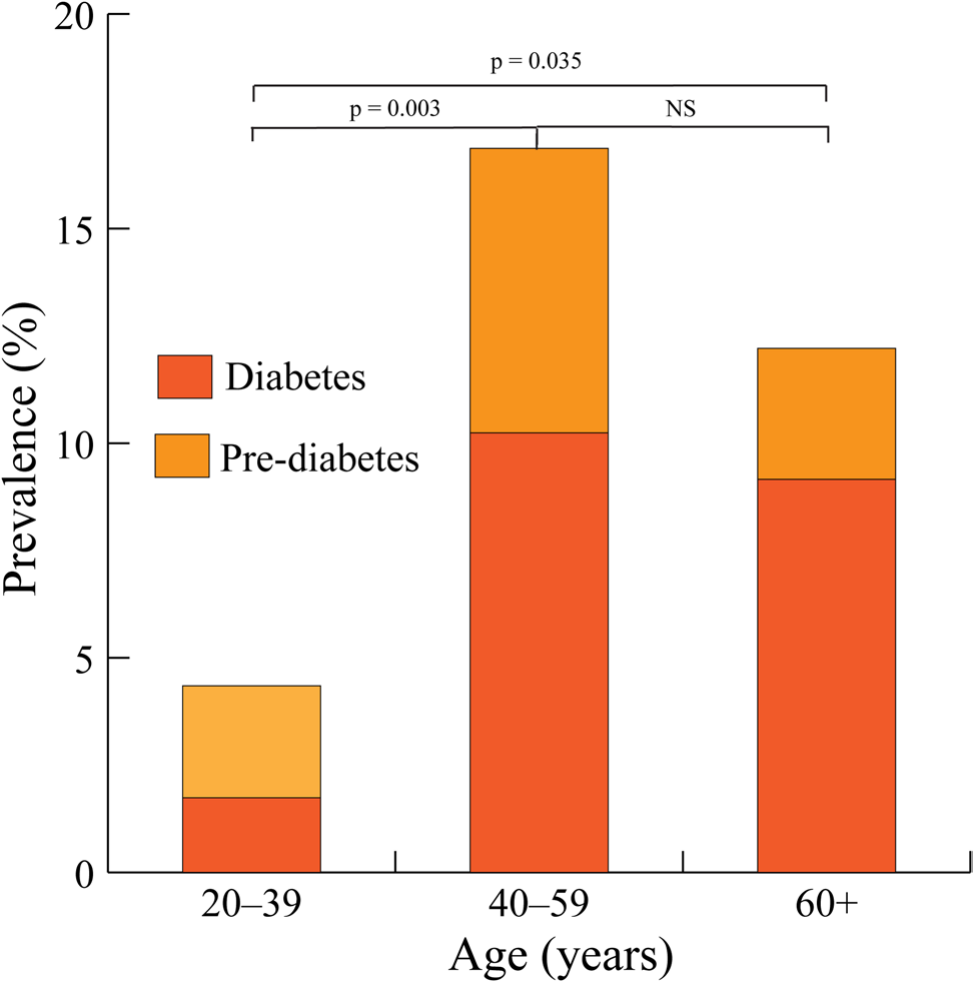
Prevalence of pre-diabetes and diabetes by subject age, Nièna, Mali, 2021. The bottoms and tops of the stacked columns represent diabetes and pre-diabetes groups, respectively. Statistically significant differences were observed for the 20–29 versus 40–49 and 30–39 versus 40–49 age group comparisons.

Approximately 31.8% (131/412) of study participants were aged 60 years old or older. Pre-diabetes was mainly observed among subjects aged between 40 and 59 years, while diabetes was most commonly observed in participants aged 40 to 59 years (61.1% [11/18] and 54.8% [17/31], respectively, Fig. 3).

Diabetes prevalence rates were 10.2% and 9.2% for participants in the 40–59 and 60+ age groups, respectively. The proportion of pre-diabetics declined with increasing age among subjects aged at least 40 years (6.6% [11/166] and 3.1% [4/131] in the 40–59, and 60+ age groups, respectively). A sharp uptick in the percentage of the sampled population testing positive for diabetes was observed in subjects aged at least 40 years (1.7% [2/115] for the 20–39 year age group versus 10.2% [17/166] for the 40–59 year age group). An uptick in the proportion of pre-diabetics was also observed in subjects aged at least 40 years (2.6% [3/115] for subjects aged 20–39 years versus 6.6% [11/166] for subjects aged 40 to 59 years). The age-by-sex distributions for the pre-diabetic and diabetic groups are provided as supplementary information (Supplemental Figs. S2, S3). A stratified (by pre-diabetic and diabetic groups) presentation of age-specific prevalence rates expressed as 10-year age groups are provided in Supplement Fig. S4.

### BMI by diabetic status

**Figure 4:**
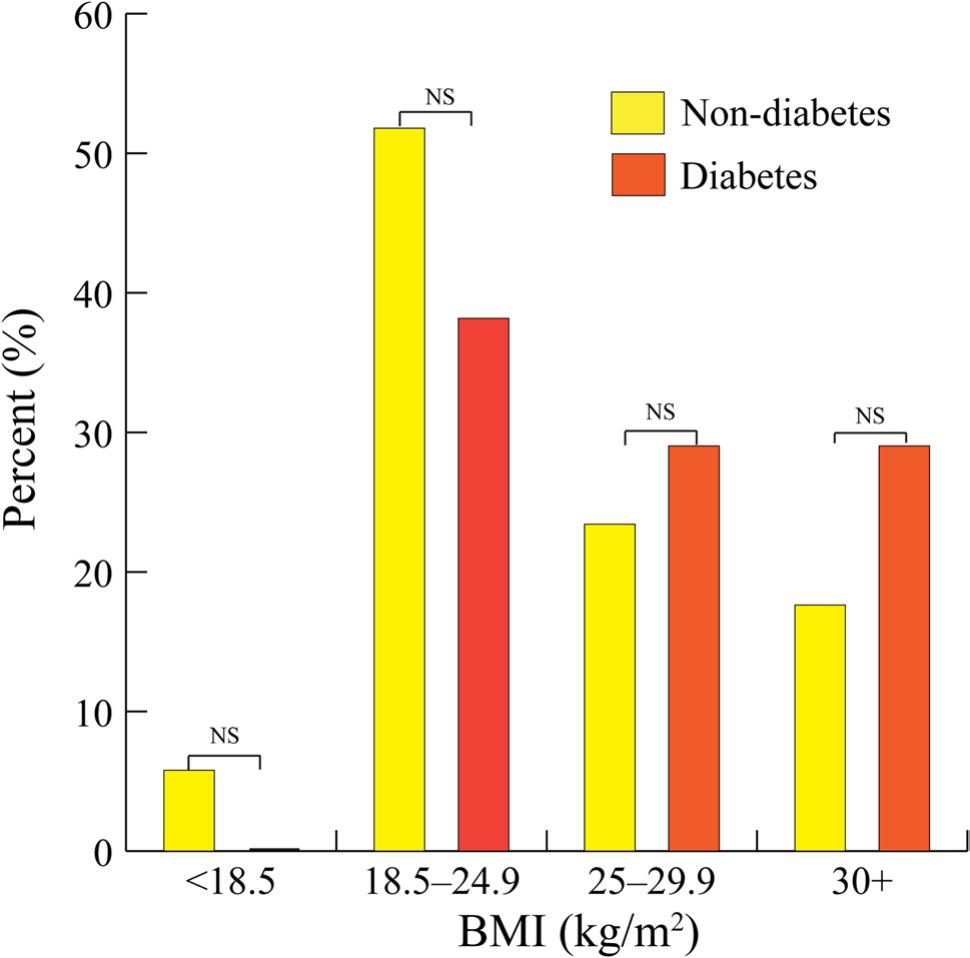
Body mass index by diabetic status, Nièna, Mali, 2021. The first and second columns show the percentage of non-diabetes and diabetes subjects for each body mass index (BMI) classification. The percentages of non-diabetic and diabetic subjects did not statistically differ within the BMI classifications.

Overall, 19.7% (80/406) of subjects had a BMI of at least 30.0 kg/m^2^, which was the threshold for overweight status. Approximately 5.4% (22/406) of subjects were underweight (less than 18.5 kg/m^2^). No underweight participants were observed in the diabetes group (0.0%, [0/30]). Approximately 5.9% [21/358] of non-diabetic subjects were underweight, while 30.0% [9/30, two missing BMI values] of T2D subjects were classified as overweight (Fig. 4).

BMI was statistically associated with diabetic status (p = 0.004). Interestingly, BMI was not statistically related to the diabetic group after excluding pre-diabetics (p = 0.166). However, it is worth mentioning that the percentage of diabetics was higher among subjects classified as overweight and obese. For this reason, this finding may be an artifact of the sample sizes for each diabetic classification.

### Diabetic awareness

**Figure 5:**
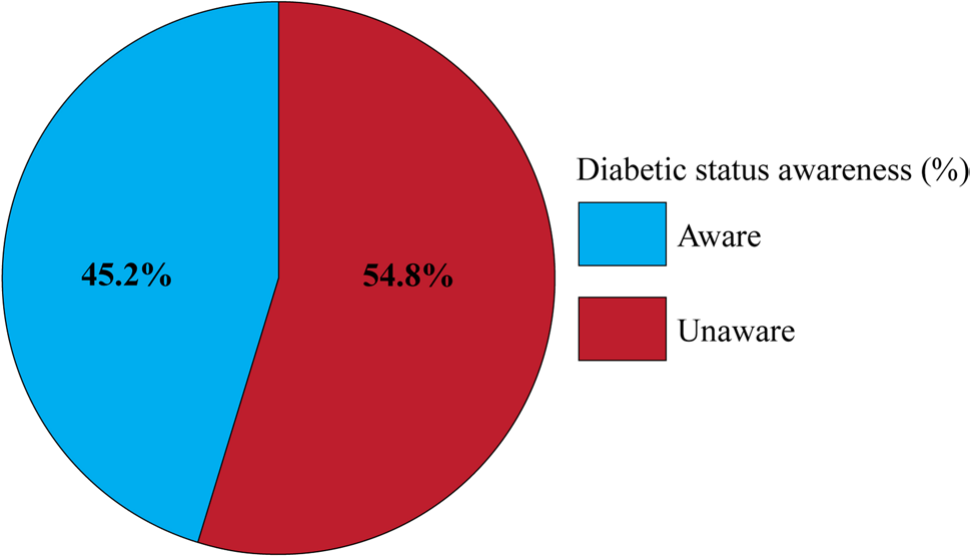
Undiagnosed diabetics among rural residents over 20 years old in Nièna municipality. Among T2D participants, 54.8% (17/31) of subjects that tested positive for diabetes were unaware of their diabetic status.

Selected subjects were also asked whether they currently have diabetes. Among subjects testing positive for T2D, 61.3% (19/31) indicated that they do not currently have diabetes, suggesting that these subjects were of their diabetic status before the screening performed in this study (Fig. 5).

### Prevalence of fetal macrosomia

Female participants were asked whether they previously had any newborn deliveries weighing over 4 kg, which were classified as macrosomic pregnancies. A total of 31 (12.6%) non-T2D female participants and 8 (34.8%) T2D participants reported experiencing at least one newborn delivery over 4 kg. Thee differences were significantly different (p < 0.001). More specifically, T2D participants were 3.70 times more likely to have at least one newborn weighing over 4 kg than non-T2D subjects (95% CI 1.45, 9.44).

### Multivariable analyses

Bivariate analyses revealed that a family history of T2D and hypertension were significantly associated with T2D (Table 1). Multivariable logistic regression analyses were performed modeling T2D against these two predictors. These predictors were jointly associated with T2D (Table 2).

**Table 2:** Factors associated with T2D using multivariable logistic regression models.

Risk factor	OR	95% CI	p value
Age^1^			
40‒59	5.22	1.09, 25.03	0.073
≥ 60	4.44	0.89, 25.12	0.208
Family history of diabetes^2^	14.13	6.10, 32.75	< 0.001
Hypertension^3^	2.06	0.86, 4.95	0.106
High waist circumference^4^	2.07	0.75, 5.71	0.160

*OR* odds ratio, *CI* confidence interval, *T2D* type 2 diabetes.

^1^Reference group = subjects aged 20‒39 years.

^2^Based on immediate family members (father, mother, sister, or brother). Reference group = no family history of diabetes.

^3^Classified as hypertensive (prior diagnosis of hypertension or systolic blood pressure greater than or equal to 140 mmHg or diastolic blood pressure of at least 90 mmHg or use of antihypertensive drugs). Reference group = non-hypertensive.

^4^Normal defined as less than 80 cm and 90 cm for females and males, respectively. High was defined as at least 80 cm and 90 cm for females and males, respectively. Reference group = normal waist circumference.

Subjects 40‒59 years old were 5.22 times more likely to have a positive T2D diagnosis than subjects in the 20‒39 age group (95% CI 1.09, 25.03). Those subjects with a family history of diabetes were 14.13 times more likely to have T2D than those without a family history of diabetes (95% CI 6.10, 32.75). Hypertension status was not significantly associated with T2D status after controlling for age group, family history of diabetes, and waist circumference (95% CI 0.86, 4.95). Waist circumference was not related to T2D status after controlling for age group, family history of diabetes, and hypertension (95% CI 0.75, 5.71).

## Discussion

This study revealed that the prevalence of T2D is likely higher than previously reported in Mali. The IDF recently reported T2D prevalence among adults aged 20 to 79 years in Mali at 1.8% (95% confidence interval 1.3%, 2.8%)^1^. It is noteworthy that the upper confidence limit for this estimate was less than half of the overall 7.5% T2D prevalence observed in this study. These differences may be partially influenced by urbanicity in the IDF estimates and underreporting. The findings in this study support the notion that T2D is highly prevalent in rural Malian communities, which is consistent with other studies reporting T2D as an emerging crisis in West Africa^9^. It has been established that diabetes prevalence is rising more rapidly in low- and middle-income countries than in more developed countries^26^. Still, it remains largely unknown whether the current T2D situation in rural Mali is directly related to improved detection, population aging, or the mitigation of competing infectious diseases. The enhanced detection explanation is supported by early studies dating back to the 1970s, which suggested that T2D likely occurred in up to 10% of Mali’s population^20^.

### Age and BMI

Observed T2D prevalence rates were lowest in the 20‒39 year age group, which was consistent with other studies reporting that T2D onset typically occurs in middle-aged individuals^27^. The age distribution for TD2 participants in the 40‒59 year age group here was slightly higher than those in the same age range reported for the African Region^28^ (53.0% here versus 43.2% for the African Region, respectively). Pre-diabetes was more frequently associated with higher BMI than confirmed T2D, but these differences may be an artifact of the small sample sizes over the BMI strata.

### Diabetic awareness and control

Notably, a majority of participants meeting the definition of T2D were unaware of their diabetic status before the study screenings. For this reason, this study served to jointly quantify T2D occurrence while raising awareness and improving education about T2D in the Nièna community. The IDF recently reported that 87.8 per 1000 adults aged between 20 and 79 years are undiagnosed in Mali, which over one half of the 152.5 per 1000 adults with T2D^1^. Limited data for this study (not shown) suggested that control measures for T2D subjects in rural Mali were also highly deficient, which may be related to access to treatment options and awareness about potential T2D complications.

### Health seeking behavior

Participants were predominantly female, which we believe was primarily due to more active participation rates among females. In light of the COVID-19 pandemic, health-seeking behaviors have changed, resulting in lower presentation rates to clinics for routine care^29^. While the study was conducted during the COVID-19 pandemic, the impact of health-seeking behavior changes due to the pandemic was beyond the scope of the study. Prior studies in Mali have suggested that internal conflict impacts the care and treatment of diabetes^30^. Because this study was performed in the community, health-seeking behavior and travel barriers may have been less influential than in studies conducted at large hospitals in urban settings. While the study did not assess longitudinal patterns over time, we believe that the high prevalence rates here are not a recent phenomenon.

### Diabetes risk factors

T2D was associated with hypertension and a family history of diabetes and primarily occurred in middle-aged participants, consistent with other studies^31^. T2D increases were more discernable among females than males, which may be partly attributable to the larger sample of female participants. T2D symptoms may take years to develop and be prolonged due to a lack of detection, particularly for asymptomatic subjects. Because the effects of T2D may not manifest as noticeable symptoms, screening based on quantitative measures known to be associated with T2D is needed. It has been broadly established that high blood pressure is common in T2D subjects, which was supported by the findings in this study. This study also revealed a strong link between T2D and the familial history of T2D, which highlights the impact of raising awareness of T2D status within families.

### Fetal macrosomia

It has been reported that fetal macrosomia may occur in 12% of newborns in the general population and 15–45% of mothers with gestational diabetes mellitus (GDM)^32^. In this study, T2D women were considerably more likely to encounter fetal macrosomia deliveries than non-T2D women. While GDM was not measured in this study, the 12.6% of non-T2D participants reporting a newborn delivery over 4 kg is consistent with the general population and may indicate that GDM rates (among non-T2D females) are not extreme in this population.

### Strengths and limitations

A particular strength of this study is that, to our knowledge, it is the first of its kind to characterize diabetes prevalence and its risk factors in the rural community of Nièna, Mali. This study employed active case detection, which perhaps is less susceptible to self-presentation bias than studies performed at large hospitals in urban areas. The study also had several limitations. The sample size included a relatively small sample of 31 T2D subjects. Studies focusing on larger numbers of T2D participants or T2D-specific studies are needed to adequately assess subgroup comparisons in T2D rural African populations. Also, T2D was measured using fasting blood sugar or prior T2D diagnosis, which is perhaps less reliable than confirmatory tests such as oral glucose tolerance tests. Longitudinal FBS tests or glycated hemoglobin tests (HBa1C levels) are needed to assess the current status of diabetic treatment and control, which was beyond the scope of this study.

## Conclusion

Field studies play a crucial role in accentuating diabetes prevalence and raise awareness, particularly in rural areas in low-resource settings where routine testing is not routinely performed. Knowledge about T2D prevalence in rural areas where data systems are often weak is a step toward improving resource allocation and targeted prevention strategies. The recent studies showing the crisis of T2D in Africa and the results in this work highlight the urgency for increased diabetic awareness and control measures. The findings here provide a resource to quantify, understand, treat, and mitigate T2D in rural Mali and, more broadly, in West Africa.

## Supplementary Information

Supplementary Figures.

## Data Availability

Data for this study are provided within the article and tabulated in Table 1.

## Abbreviations

BMI: Body mass index
COVID-19: Coronavirus disease 2019
GDM: Gestational diabetes mellitus
IDF: International Diabetes Federation
NCD: Noncommunicable disease
OR: Odds ratio
SSWA: Sub-Saharan West Africa
T2D: Type 2 diabetes
WHO: World Health Organization
