# Supplementary material for: Type 2 diabetes prevalence, awareness, and risk factors in rural Mali: a cross-sectional study
Source: Sci Rep. 2023 Mar 6;13:3718. doi: 10.1038/s41598-023-29743-1 (PMC9987397; doi:10.1038/s41598-023-29743-1)

**Supplementary Information**

**Supplemental Figure S1.** Study flow chart and analytical flow. Multivariate analyses were performed on study variables associated with diabetic status using bivariate analyses.


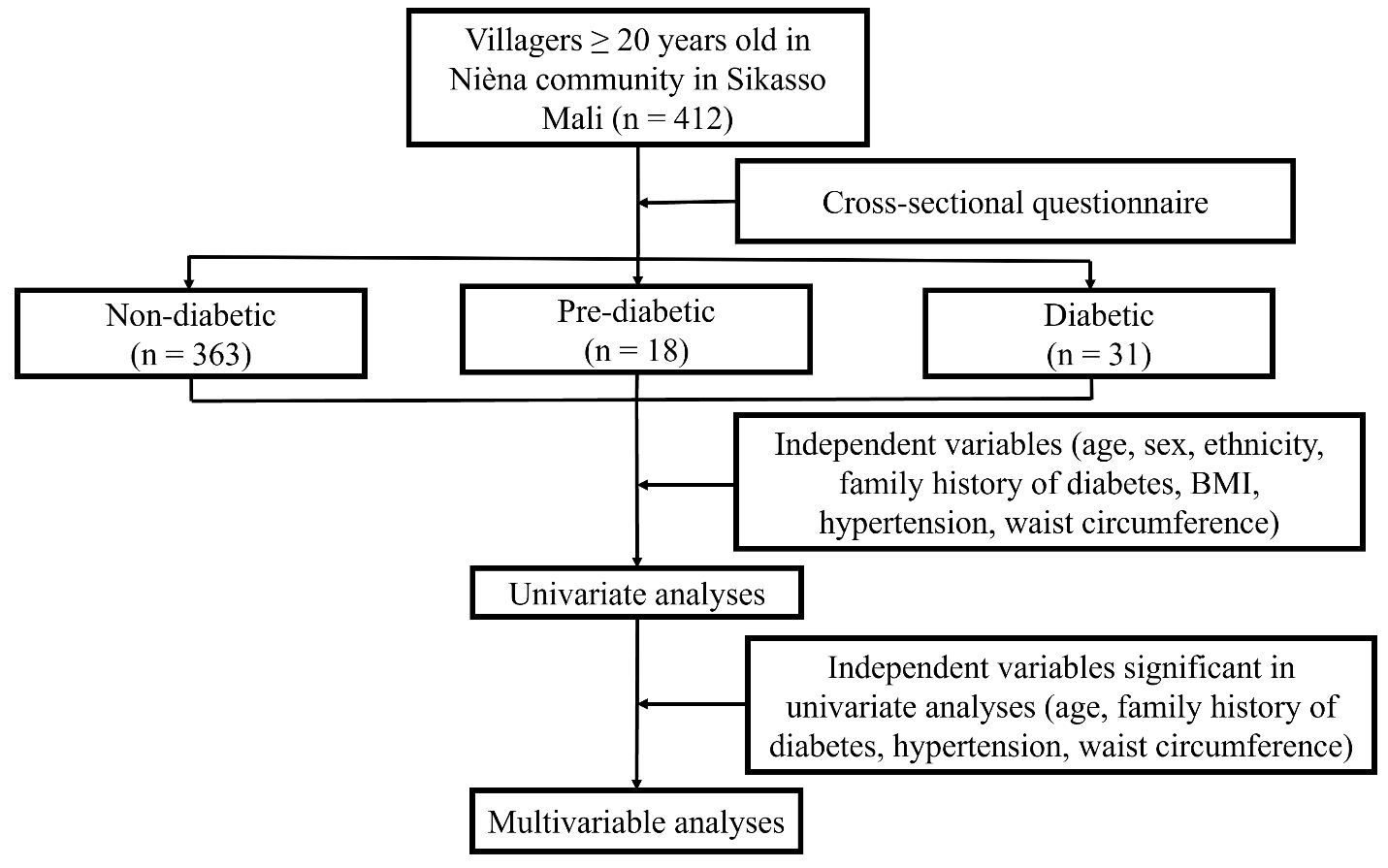


**Supplemental Figure S2 (corresponds to Figure 3)**. Age distribution for n = 18 subjects classified as pre-diabetic in Nièna municipality, 2021. All study participants were aged 20 years or older. No pre-diabetics were observed for the 20–29 year age group.


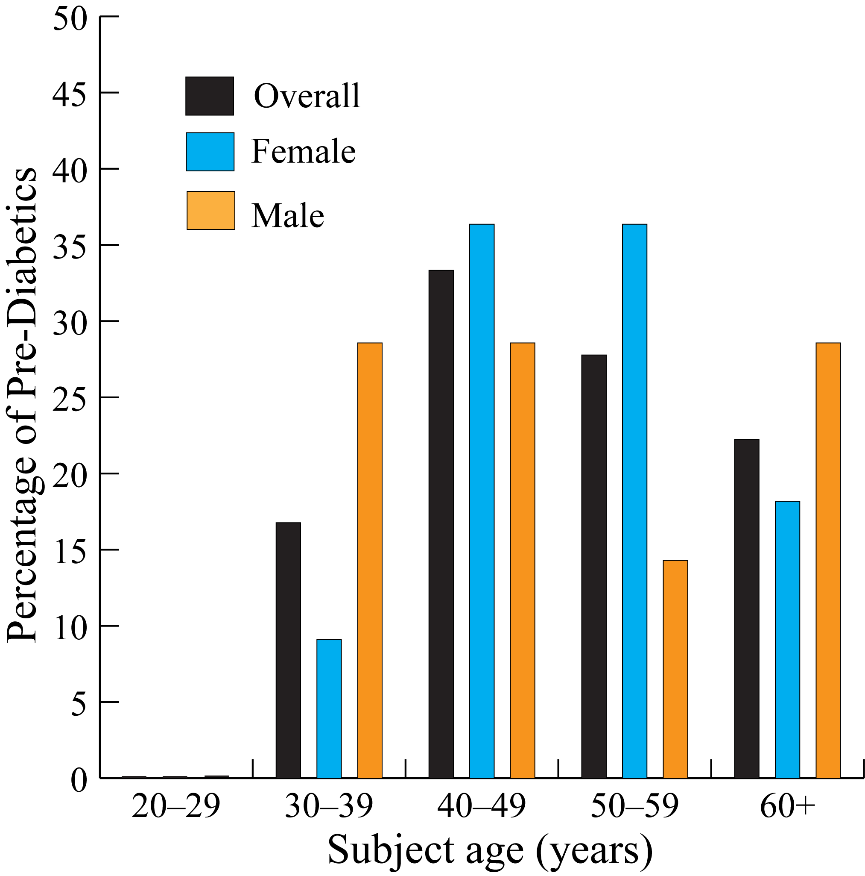


The first, second, and third columns show the prevalence rates for the overall population, females, and males in Niena, Mali, respectively.

**Supplemental Figure S3 (Corresponds to Figure 3)**. Age distribution of n = 31 subjects classified as diabetic among n = 412 residents in Nièna municipality, Mali, 2021. All study participants were aged 20 years or older. The first, second, and third columns show the prevalence rates for the overall population, females, and males in Niena, Mali, respectively.


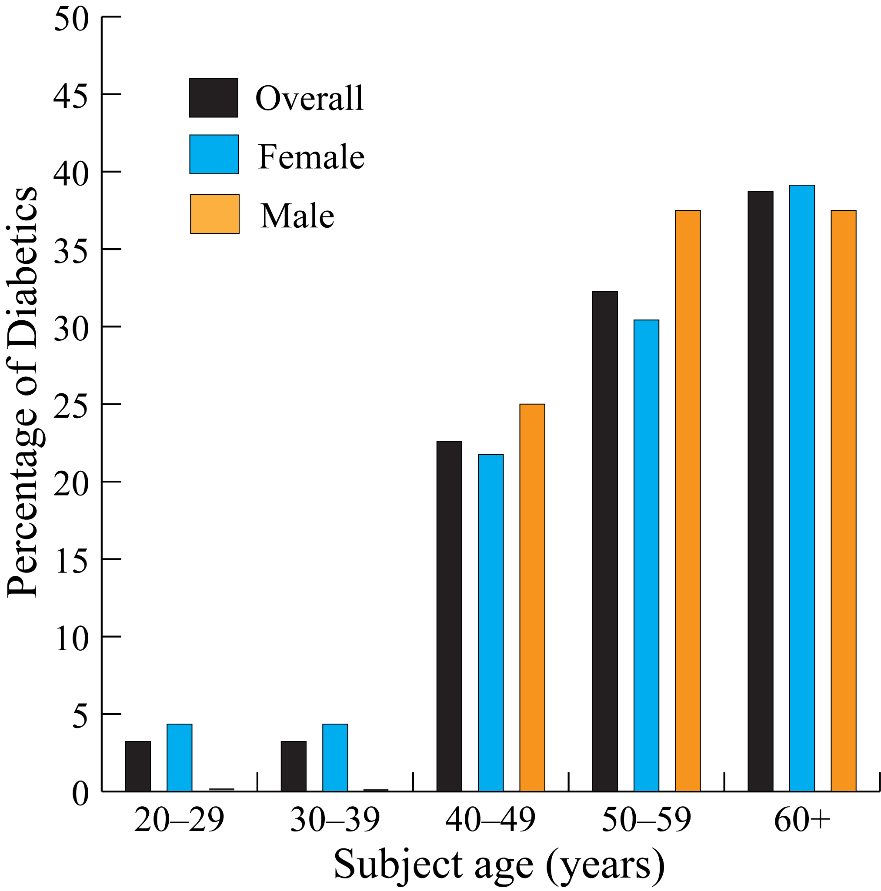


**Supplemental Figure S4 (Corresponds to Figure 3)**. Observed pre-diabetics (n = 18) and diabetics (n = 31) by age group among n = 412 residents over 20 years old in Nièna municipality, 2021.


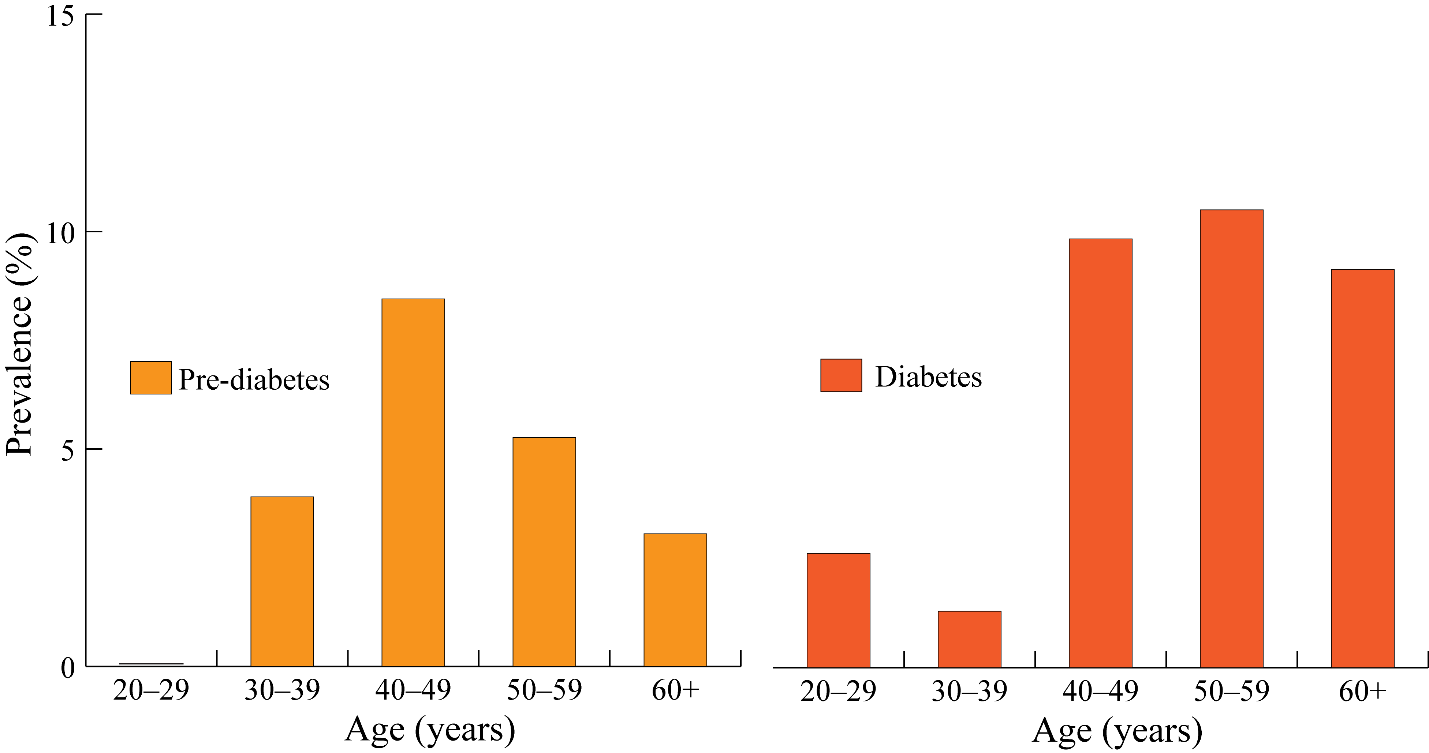

Supplement: Supplementary file 1 — Supplementary Figures. [file 41598_2023_29743_MOESM1_ESM.docx]
